# Effect of Dual-Modified Tapioca Starch/Chitosan/SiO_2_ Coating Loaded with Clove Essential Oil Nanoemulsion on Postharvest Quality of Green Grapes

**DOI:** 10.3390/foods13233735

**Published:** 2024-11-22

**Authors:** Hui Chang, Kaimian Li, Jianqiu Ye, Jian Chen, Jie Zhang

**Affiliations:** 1 Tropical Crops Genetic Resources Institute, Chinese Academy of Tropical Agricultural Sciences, Haikou 571101, China; changhui199907@163.com (H.C.); likaimian@sohu.com (K.L.); yejianqiu2006@126.com (J.Y.); 2 Key Laboratory of Food Nutrition and Functional Food of Hainan Province, Engineering Research Center of Utilization of Tropical Polysaccharide Resources, Ministry of Education, College of Food Science and Engineering, Hainan University, Haikou 570228, China

**Keywords:** tapioca starch, clove essential oil, fruit preservation, starch-based coating

## Abstract

As consumer awareness regarding health and nutrition continues to increase, there is a growing demand for fresh, nutritious fruits such as green grapes. However, the short storage life and susceptibility of these fruits to spoilage lead to significant commercial losses. Currently, the plastic wrap method is commonly used to keep green grapes fresh, but this packaging effect is limited and not environmentally friendly. Therefore, there is an urgent need to explore sustainable and effective preservation methods. In this study, a high-pressure microfluidization technique was employed to prepare an essential oil nanoemulsion with a ratio of Tween 80 to clove essential oil of 1:1, and a biopolymer-based film solution was prepared using dual-modified tapioca starch and chitosan loaded with clove essential oil nanoemulsion. The dual-modified tapioca starch/chitosan/SiO_2_/1.25 wt % clove essential oil (DM/Ceo-1.25) solution coating was successfully applied for the packaging and preservation of fresh green grapes. Compared with the CK and polyethylene wrap (PE) groups, the DM/Ceo-1.25 coating significantly improved the quality of the green grapes, increasing the storage period of the green grapes from 4 to 8 days at room temperature. On the 10th day of storage, the coated grapes retained significantly better quality, with a hardness of 4.01 N, a titratable acidity of 1.625%, an anthocyanin content of 1.013 mg/kg, and a polyphenol content of 21.32 μg/mL. These results indicate that the DM/Ceo-1.25 solution coating developed in this study can be used as a new active material for fruit preservation and provides ideas for the development of safer and more sustainable food packaging.

## 1. Introduction

Extending the shelf life of food is a crucial area of research within modern food science and technology, as it not only helps reduce food waste but also preserves the nutritional value of food. The traditional fruit preservation methods generally involve plastic packaging, but the raw materials of plastic are petroleum-based, which can easily cause white pollution. With advancements in science and technology, the adoption of green methods for prolonging food shelf life has become especially important. These methods often involve the utilization of natural preservatives, bio-based coating packaging and other methods [1].

Among these methods, active edible coatings are effective in preserving various fruits. Green grapes are a type of fruit with high nutritional value. They contain abundant substances such as anthocyanins and total phenols, which contribute to their nutritional value and health benefits. In addition, soluble solid content and titratable acidity are important quality indicators for further evaluating green grapes. Owing to their high sugar content and high content of active substances, they are susceptible to microbial contamination and damage, which severely impacts their safety, quality, and shelf life [2]. Considering the high respiration rate of green grapes, coating solutions can be considered effective packaging protection: they can form a protective layer on the surface of a fruit, regulate the respiration rate of the fruit, control the loss of water, and thus reduce the occurrence of spoilage.

One of the commonly used film-forming substances in coating technology is tapioca starch, which is widely utilized as a film-forming material owing to its high flexibility and high transparency after gelatinization. However, the low solubility and high viscosity of natural tapioca starch limit its practical application [3]. The dual-modified tapioca starch obtained after citric acid and ultrasonic modification can be better mixed with other materials to form composite films [4].

Glycerol is a commonly used plasticizer in film preparation. Previous studies have shown that when glycerol is used as the sole plasticizer blended with starch, the plasticizing effect is weak, adversely affecting the film-forming ability of the starch and resulting in insufficient uniformity and integrity of the film [5]. This can lead to increased brittleness during use, thereby impacting its mechanical properties. Therefore, incorporating other natural polymers, such as chitosan, gelatin, or Arabic gum, into composite films may be helpful for enhancing their flexibility and water resistance [6].

Chitosan (CS) is a natural and edible polymer that is both nontoxic and eco-friendly. It is widely recognized as a versatile biopolymer and is often employed as an edible coating for fruits and vegetables owing to its superior ability to form films and its biocompatibility [7]. Recent studies have shown that the incorporation of CS can increase the mechanical strength and thermal stability of films [8]. Tapioca starch-based composite films are typically brittle. The addition of chitosan significantly improved the ductility and tensile strength of the composite films. This enhancement increases the durability of the films in practical applications, particularly in the area of packaging materials.

The nano-SiO_2_ possesses characteristics of light transmission and a small particle size. The research by Liu et al. reported that these properties can increase the density and strength of composite materials [9]. In addition, the incorporation of nano-SiO_2_ has the potential to enhance the barrier properties of the coating, effectively reducing the permeation of oxygen and moisture [10]. We chose to combine chitosan with nano-SiO_2_ as a reinforcing agent and blend it with dual-modified tapioca starch to produce a film matrix.

Despite the excellent film-forming and barrier-enhancing properties of CS and nano-SiO_2_, the incorporation of an effective antibacterial agent is crucial to further improve the antimicrobial effectiveness of these coatings. To overcome this limitation, the addition of an effective antibacterial agent is needed.

Clove essential oil (Ceo) is a natural substance derived from plants. Previous studies have shown that Ceo emulsions have excellent inhibitory effects on the growth of foodborne pathogens [11]. Nimesh Dileesha Lakshan et al. successfully incorporated Ceo emulsions into arrowroot starch, resulting in an edible coating that significantly improved the postharvest quality of tomatoes [12]. Previous studies have also shown that the incorporation of ultrasonically treated nanoemulsions into pullulan–sodium alginate films resulted in promising outcomes for extending the storage period of cherries [13].

However, the exceptional antimicrobial properties of essential oils (EOs) are limited by their high volatility, instability, and hydrophobicity. Nanoemulsions of essential oils are formed by dispersing essential oils at the nano scale (typically between 20 and 200 nanometers) in an aqueous phase or other liquids, creating a uniform liquid system [14]. The fabrication process of essential oil nanoemulsions can conceal the odor of essential oils, increase their solubility and stability in the aqueous phase, regulate the release rate of bioactive compounds, and improve their biological activity. To our knowledge, there is a scarcity of research on the application of Ceo in the form of nanoemulsions incorporated into bio-based films for food packaging.

Therefore, we hypothesize that the Ceo nanoemulsion, when incorporated into a film matrix primarily composed of dual-modified tapioca starch and chitosan, plays a significant role in preserving green grapes. Since previous studies have shown that chitosan coatings alone can also achieve similar preservation effects [15], we designed two experimental groups: the DM/CS (DM) coating solution group and the DM/CS/Ceo-1.25 (DM/Ceo-1.25) coating solution group to treat the fruits separately to further verify the antibacterial and preservative effects of clove oil nanoemulsions. In addition, we tested a series of quality and nutritional indicators during the storage process to compare the effects between the different groups. Additionally, our study explored the influence of different amounts of film components, such as dual-modified tapioca starch, glycerin, and SiO_2_, on the mechanical properties of the film. In conclusion, this work proposes a new method of food preservation, improves the quality of green food packaging, and reduces agricultural waste and economic losses.

## 2. Materials and Methods

### 2.1. Materials

Food-grade tapioca starch (17% apparent amylopectin) was purchased from Haining Fengyuan Food Co., Ltd. (Haining, China). Nano-SiO_2_ (hydrophilic; purity of 99.5%) was purchased from Fengrun Chemical Corporation (Ganyu, China). Tween 80 (analytical-grade nonionic surfactant; appearance: yellow to amber viscous liquid; density: 1.06 to 1.10; refractive index: 1.4756; viscosity: 0.4 to 0.7 Pa·s; flashpoint: 10 °C), clove essential oil (appearance: colorless or pale yellowish liquid; scent: aroma of cloves, strong flavor; density: 1.042; refractive index: 1.5291; flashpoint: 112 °C; eugenol content: 85.2%), chitosan (CAS No. 9012-764; MW = 50–170 KDa; degree of deacetylation = >75%) and LB (Luria–Bertani broth) medium were purchased from McLean Reagent Corporation (Shanghai, China). The plastic wrap (PE) manufacturer was MiaoJie (Jiangyin, China). Green grapes were bought from Hainan’s local fruit shop.

### 2.2. Preparation of Clove Essential Oil Emulsion

The Ceo and Tween 80 were mixed as oil phases at volume ratios of 1:2, 1:1, 2:1, 3:1, and 4:1, with deionized water as the water phase, and the mixture was homogenized at 15,000 psi for three cycles using a high-pressure homogenizer (NanoGenizer30k, Aoyi Instrument Co., Ltd., Shanghai, China), then collected and stored at 4 °C [16].

### 2.3. Characterization of Clove Essential Oil Emulsion

The size, zeta potential, and polymer dispersity index (PDI) of the emulsion were measured by a particle size potentiometer (Mastersizer 3000+ Ultra, Malvern Instruments Co., Ltd., Malvern, UK). In addition, the emulsion was stored at room temperature for 24 h to observe whether it was stratified to determine its stability. The droplet dispersion state is observed by dispersing the emulsion in deionized water to form a 30% oil/water system under an optical microscope (100×) (Axio Observer, Carl Zeiss Co., Ltd., Oberkochen, Germany) [17].

### 2.4. Preparation of Dual-Modified Tapioca Starch

Dual-modified tapioca starch was prepared by combining citric acid with ultrasonic waves. The details are as follows. Some modifications were made to the method proposed by Pornsuksomboon et al. [18]. A 40 g amount of citric acid was dissolved in 100 mL of distilled water. Subsequently, the pH of the solution was adjusted to approximately 4 using 10.0 mol/L NaOH. Afterward, this solution was mixed with 100 g of tapioca starch to obtain a uniform slurry. After storing at room temperature for 12 h, the slurry was treated with ultrasonic power at 450 W for 40 min. Upon completion of the treatment, the slurry was dried in an oven at 95 °C until a constant weight was achieved.

### 2.5. Preparation of Composite Films

Using the impact on the mechanical properties of the film as the criterion, we optimized the addition amounts of the main components and determined the following specific parameters for the preparation of a composite film containing Ceo nanoemulsion. A 2% chitosan solution was prepared by dissolving it in a 1% acetic acid solution. In 100 mL of deionized water, 2 g of dual-modified tapioca starch was dissolved and heated at 80 °C for 15 min to obtain a gelatinized state [19]. The two solutions were then mixed at a 1:1 mass ratio on a magnetic stirrer. Then, 20% db (dry basis) glycerol was added to the mixture, and the mixture was stirred for 1 h until homogeneous. Additionally, 1.25% db SiO_2_ based on dry mass was incorporated. Subsequently, nanoemulsions of clove essential oils were incorporated at different concentrations (0 wt%, 0.5 wt%, 0.75 wt%, 1.0 wt%, and 1.25 wt%), designated DM, DM/Ceo-0.5, DM/Ceo-0.75, DM/Ceo-1.0, and DM/Ceo-1.25, respectively. To fabricate a film, 60 mL of the film mixture was poured into a circular Petri dish with an area of 66.44 cm^2^. All the film solutions were dried in an oven at 45 °C and left at room temperature until they became soft enough to be peeled off. Six parallel samples were prepared for each film.

### 2.6. Mechanical Properties

The composite films were shaped into dumbbell forms measuring 1 cm in width and 3 cm in length. Mechanical properties were evaluated by performing a stretching test using a texture analyzer (WDW-6100, (WDW-6100, Suzhou Baoman Precision Instruments Co., Ltd., Suzhou, China). The test was conducted following the ISO 1184-1983 standard [20]. The film was stretched at a contact force of 5 g and a rate of 20 mm/s until the film broke.

### 2.7. Light Transmittance of Film

Light transmittance was measured by cutting the composite film into 4 × 1 cm^2^ strips. The cut samples were scanned by visible light scanning in the range of 400 to 780 nm [21].

### 2.8. Antioxidant Capacity

In total, 0.05 g of film (DM, DM/Ceo-0.5, DM/Ceo-0.75, DM/Ceo-1.0, and DM/Ceo-1.25) was weighed and soaked in 4 mL (0.01 mmol/L) of a DPPH methanol solution in the dark for 30 min. The absorbance of the solution at 517 nm was measured via a microplate reader to calculate the DPPH radical-scavenging activity of different composite film solutions. The five types of films were immersed in 5 mL of ethanol solution (0.1 mmol/L) in the dark for 24 h to obtain the film extracts [22].

The extraction solution of the film (40 μL) was then mixed uniformly with ABTS solution (100 μL) and incubated for 10 min. The absorbance of the solution at 734 nm was measured via a microplate reader to calculate the ABTS radical-scavenging activity of different composite film solutions (TU-1901, Puxi General Instrument Co., Ltd., Beijing, China). [23]. Each sample was analyzed in triplicate. The antioxidant activity was calculated using the following formula:

(1) $$\mathrm{Antioxidant}\;\mathrm{rate}\;\left(\%\right)=\frac{A_{\mathrm{Sample}}-A_{\mathrm{Blank}}}{A_{\mathrm{Sample}}}\times 100\%$$

### 2.9. Preparation of Coating Film Solutions

We prepared two types of coating solutions. A 2% dual-modified tapioca starch solution was heated at 80 °C for 30 min to achieve a gelatinized state, with a 1% chitosan solution added at a mass ratio of 1:1. Then, 20% db (dry basis) glycerol was added to the mixture, and the mixture was stirred for 1 h until homogeneous. Additionally, 1.25% db SiO_2_ based on dry mass was incorporated to prepare the DM coating solution. A 1.25 wt% Ceo nanoemulsion was added to the DM coating solution to prepare the DM/Ceo-1.25 coating solution. Both solutions were mixed uniformly at 450 rpm on a magnetic stirrer for 3 h until a smooth, sediment-free film-forming solution was obtained. The compositions of the film solutions are shown in Table 1.

### 2.10. Green Grape Preservation

The fresh green grapes were disinfected in 1% sodium hypochlorite solution for 120 s, followed by rinsing with tap water and drying at room temperature [24]. Four processing groups were set up, namely the CK group, PE group, DM group, and DM/Ceo group. In the DM group and DM/Ceo group, the green grapes were immersed in the coating film solution for 60 s, removed and hung on a cotton thread to dry at a constant temperature (25 ± 1 °C, 85–90% RH). This process was repeated three times, and the coating film solution was thoroughly dried and collected. The quality index was determined at 0 d, 2 d, 4 d, 6 d, 8 d, and 10 d.

CK group: Washed with deionized water and dried.

PE group: Wrapped with commercially available plastic wrap.

DM group: Coated with DM film solution.

DM/Ceo group: Coated with DM/Ceo film solution.

#### 2.10.1. Water Contact Angle of Coating

After the peels of the CK group and the two coating groups (DM and DM/Ceo groups) were allowed to dry completely, square pieces of peels measuring 1 × 1 cm from the fruits of the three groups were carefully cut. The grape peel samples were then mounted on slides, and a drop (3.0 μL) of deionized water was placed on the surface of each sample. After deposition, images of the water or coating solution droplets were immediately captured, and the hydrophobicity of the coatings was analyzed via an interface tensiometer [25]. The water contact angle is the angle formed between the surface of a liquid droplet and the solid surface when the droplet is placed on the solid. A contact angle of less than 90° indicates that the material is easily wetted by the liquid. A contact angle greater than 90° suggests that the material is not easily wetted by the liquid. A contact angle greater than 150° indicates that the material is extremely hydrophobic [26].

#### 2.10.2. Scanning Electron Microscopy of Peels

After the coated green grapes were completely dried, a knife was used to carefully cut squares of fruit peels measuring 0.5 cm in width and 0.5 cm in length and the morphology of the peels was observed with a field emission scanning electron microscope (Verios G4, Thermo Fisher Scientific Co., Ltd., Glasgow, UK) [27].

#### 2.10.3. Microbiological Analysis

Samples from the four different treatment groups (CK group, PE group, DM group, and DM/Ceo group) were accurately weighed at 2 g on days 0, 2, 4, 6, 8, and 10, and evenly dispersed in 18 mL of sterile saline for gradient dilution. Then, 200 μL of the 10^−5^ dilution was evenly spread on potato dextrose agar (PDA) plates and incubated at 36 °C ± 1 °C for 48 ± 2 h [28].

#### 2.10.4. Color Measurement

At 0 d, 2 d, 4 d, 6 d, 8 d, and 10 d, five fruits were randomly selected from each treatment group to determine their color characteristics using a colorimeter (WF28, Aoyi Instrument Co., Ltd., Shanghai, China) [29].

#### 2.10.5. Hardness

A texture analyzer (TA.XT PlusC, Stable Micro Systems Co., Ltd., Godalming, UK) was used to assess the firmness of green grapes by puncturing three positions on each grape surface using a piercing probe. The test parameters included a puncture depth of 6 mm, a puncture speed of 1 mm/s, and a trigger force of 5 g (measured in Newtons, N) [30]. For each treatment group, 10 whole green grapes were randomly selected for measurement to ensure a representative sample size.

#### 2.10.6. Weight Loss Rate

At 0 d, 2 d, 4 d, 6 d, 8 d, and 10 d, the green grapes of different treatment groups were weighed, and the weight loss rate was calculated as follows [31]:

(2) $$\mathrm{Weight}\;\mathrm{loss}\;\mathrm{rate}\;\left(\%\right)=\frac{\mathrm{Initial}\;\mathrm{weight}-\mathrm{Weight}\;\mathrm{on}\;\mathrm{sampling}\;\mathrm{date}}{\mathrm{Initial}\;\mathrm{weight}}\times 100\%$$

#### 2.10.7. Rot Rate

The decay rate was determined based on observations of the surface conditions of the coated fruit. There are four levels of spoilage: no spoilage, grade 0; 0–1/4 spoilage, grade 1; 1/4–1/2 spoilage, grade 2; 1/2–whole-fruit spoilage, grade 3 [32].

(3) $$\mathrm{Rot}\;\mathrm{rate}\;\left(\%\right)=\sum \;\frac{\mathrm{Rot}\;\mathrm{grade}\;\times \;\mathrm{Number}\;\mathrm{of}\;\mathrm{fruits}\;\mathrm{at}\;\mathrm{that}\;\mathrm{level}}{\mathrm{Highest}\;\mathrm{rot}\;\mathrm{grade}\;\times \mathrm{Total}\;\mathrm{number}\;\mathrm{of}\;\mathrm{fruits}}\times 100$$

#### 2.10.8. Soluble Solids Content

The soluble solids content of green grapes was determined using a digital refractometer (PAL-HIKARi 5, Aoyi Instrument Co., Ltd., Shanghai, China) over different storage days [33]. After juicing the grapes on different storage days and filtering appropriately, the liquid was dropped into the sample cell of a digital refractometer to directly read the refractive index of the liquid, with the unit being °Brix.

#### 2.10.9. Titratable Acid (TA)

Different groups of 5 g (m) of experimental fruit were weighed and blended to create a homogenate, which was then diluted to a final volume of 15 mL with distilled water. The mixture was heated in a water bath at 85 °C for 40 min. After rapid cooling, the mixture was filtered and diluted to 25 mL with distilled water (V1). A 10 mL (V2) sample solution from different treatment groups was placed in a conical flask, 3 drops of phenolphthalein were added, and the mixture was titrated to the endpoint with 0.1 mol/L sodium hydroxide solution [34]. The TA is calculated as follows:

(4) $$\mathrm{TA}\;\left(\%\right)=\frac{C_{\mathrm{NaOH}}\times {\;V}_{\mathrm{NaOH}}\times M_{\mathrm{Citric}\;\mathrm{acid}}}{\frac{V2}{V1}\times m\times 1000}\times 100\%$$

#### 2.10.10. Anthocyanins, Total Flavonoid Content, Total Polyphenol Content

A total of 20 mL of acidified methanol (70% methanol and 7% acetic acid) was added to 2 g of grape homogenate that had been ground in advance, stirred, and incubated at 150 rpm under dark conditions for 1 h. The mixture was subsequently centrifuged at 4000× *g* for 20 min. The supernatant was collected, and the absorbance was measured at 529 nm and 650 nm [35]. The calculation formula is as follows:

(5) $$\mathrm{Anthocyanins}\;\left(\mathrm{mg}/\mathrm{kg}\right)=A_{529\mathrm{nm}}-0.288\times A_{650\mathrm{nm}}\times 207.247$$

The total flavone content of different samples was calculated by reading the absorbance at 325 nm, and the calculation formula is as follows:

(6) $$\mathrm{Total}\;\mathrm{flavone}\;\mathrm{content}\;=\frac{A_{325\mathrm{nm}}\times V\times N}{m}$$

V represents the extract volume, N is the dilution ratio, and m is the sample mass.

The 0.1 mL juice was diluted with 1.9 mL of distilled water, and then mixed with 1 mL of Folin–Ciocalteu reagent and 1.5 mL of Na_2_CO_3_ solution [36]. The absorbance of the solution was measured at 760 nm. The phenolic content was measured as μg/mL gallic acid.

#### 2.10.11. Sensory Evaluation

All of the samples were evaluated by 10 members of the sensory team at 0 d, 2 d, 4 d, 6 d, 8 d, and 10 d. The evaluation items were color, taste, aroma, and shape, with a total score of 100, representing 25 for each item [37]. The Sensory Evaluation Scoring Criteria for Green Grapes are as follows:

1. Color (Total: 25 points)

0–5 points: Dull color, uneven, with noticeable spots or discoloration.

6–10 points: Darker color, slightly uneven, with minor defects.

11–15 points: Mostly even color, with few blemishes.

16–20 points: Vibrant, even color, with no noticeable defects.

21–25 points: Extremely vibrant and uniform color, very visually appealing.

2. Taste (Total: 25 points)

0–5 points: Poor taste, overly sour or bland.

6–10 points: Single-note flavor, lacking sweetness or overly sweet.

11–15 points: Average flavor, balanced sweetness and acidity, but lacking depth.

16–20 points: Good flavor, pleasant balance of sweetness and acidity.

21–25 points: Rich flavor, with distinct layers and excellent mouthfeel.

3. Aroma (Total: 25 points)

0–5 points: No aroma or off-putting odor, unpleasant scent.

6–10 points: Weak aroma, slightly unpleasant or off-putting.

11–15 points: Moderate aroma, mostly natural but lacks appeal.

16–20 points: Strong aroma, fresh and natural, pleasing to the senses.

21–25 points: Extremely rich and complex aroma, intoxicating.

4. Shape (Total: 25 points)

0–5 points: Deformed shape, with noticeable defects.

6–10 points: Irregular shape, with some blemishes.

11–15 points: Generally normal shape, slightly irregular.

16–20 points: Regular shape, visually appealing.

21–25 points: Perfect shape, extremely attractive appearance.

#### 2.10.12. Pearson Correlation Analysis

Pearson correlation analysis was conducted via SPSS Statistics 20 software to examine the relationships between different preservation indicators of green grapes.

A correlation plot was employed to visualize the correlations between variables. The correlation plot presents the strength and direction of the correlation coefficients through differently colored squares. The intensity of the colors in the plot reflects the strength of the correlations between variables, whereas the numerical values represent the corresponding correlation coefficients [27].

#### 2.10.13. Statistical Analysis

The average value of the data in this study was obtained by conducting each experiment three times in parallel and is expressed as the mean ± standard deviation. SPSS and Origin 2021 software were used for statistical analysis and mapping.

## 3. Results and Discussion

### 3.1. Characterization of Clove Essential Oil Emulsion

**Figure 1:**
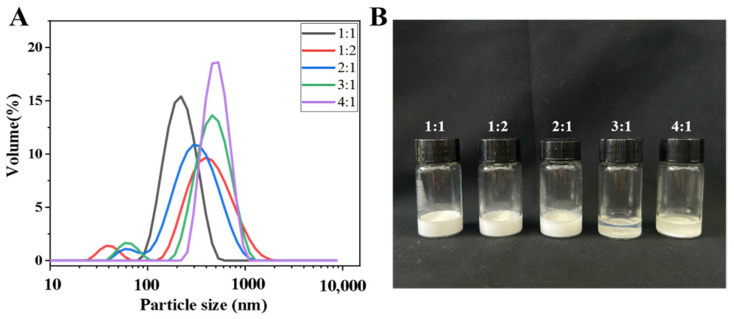

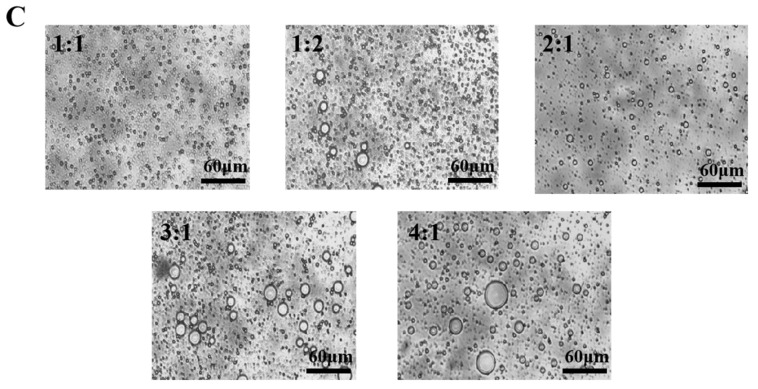
(**A**) The particle size distribution of different proportions of Ceo and Tween 80 emulsions (1:1, 1:2, 2:1, 3:1, 4:1). (**B**) The stability of different emulsions after standing at room temperature for 24 h. (**C**) Image of different proportions of Ceo and Tween 80 emulsions (30 wt%) under a bright-field microscope (100×).

The size and distribution in an emulsion are key factors in assessing its stability. The particle size distributions of the Tween 80 emulsions are shown in Figure 1A. When the essential oil/Tween 80 emulsion ratio was 1:1, the average particle size of the emulsion was 190.14 nm, which belongs to an oil-in-water nanoemulsion in the range of 20–200 nm, and the system was relatively stable, which is consistent with the stability results (Figure 1B) [38].

In Figure 1B, after 24 h of static treatment, the emulsion with 1:1 clove essential oil/Tween 80 did not stratify, whereas the emulsions with 3:1 and 4:1 clove essential oil/Tween 80 ratios were significantly stratified, indicating that these systems were unstable and that water–oil separation occurred. This result is also consistent with the optical microscope image in Figure 1C, which shows that the emulsion with clove essential oil/Tween 80 = 1:1 has a uniform droplet size and uniform distribution, whereas the emulsions with 3:1 and 4:1 have uneven droplet size distributions and easily flocculate. These results are consistent with the findings of Lim et al., who reported that the experimental group with the smallest droplet size and the most uniform droplets presented the highest stability, with no significant phase separation observed during the resting experiment [39].

The polydispersity index (PDI) can be used to reflect the uniformity of the particle size distribution of Ceo droplets. The closer the PDI value to 0, the more uniform the particle size distribution of the emulsion. As shown in Table 2, when the clove essential oil/Tween 80 emulsion ratio was 1:1, the PDI value was the smallest, at 0.188. When the Tween 80 emulsion ratio was 1:2, the PDI value was the largest, 0.511, because the excess surfactant reduced the ratio of the oil phase to the water phase, which led to collision and binding between the droplets, resulting in a significant increase in the PDI (*p* < 0.05).

In addition, the zeta potential is an important parameter that measures the magnitude of electrostatic or charge repulsion/attraction between particles in a dispersion. There is electrostatic attraction between droplets, and the greater the absolute value of the zeta potential (≥25 mV), the more stable the emulsion system [40]. Conversely, a low zeta potential can result in aggregation and destabilization of the emulsion. Among the five emulsion systems with different proportions, the absolute value of the zeta potential of the clove essential oil/Tween 80 emulsion with a ratio of 1:1 is the largest, at 32.3 mV, indicating that this system is the most stable.

In summary, a clove essential oil/Tween 80 = 1:1 emulsion was loaded into the coating film solution for subsequent experiments.

### 3.2. Effects of Different Dual-Modified Tapioca Starch Concentrations, Glycerol Contents, and SiO_2_ Contents on the Mechanical Properties of the Film

**Figure 2:**
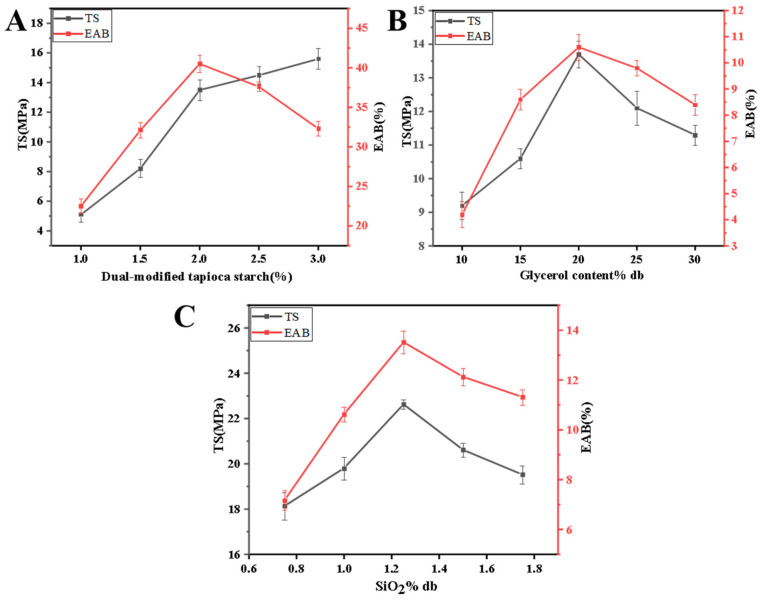
Effect of dual-modified tapioca starch, glycerol, and SiO_2_ content on the TS and EAB of composite films. (**A**) Reaction conditions: amount of glycerol: 20% db; amount of SiO_2_: 1.25% db. (**B**) Reaction conditions: amount of dual-modified tapioca starch: 2%; amount of SiO_2_: 1.25% db. (**C**) Reaction conditions: amount of dual-modified tapioca starch: 2%; amount of glycerol: 20% db. db represents dry basis.

Figure 2A–C show the effects of different dual-modified tapioca starch contents, glycerol contents, and SiO_2_ contents on the mechanical properties of the films. Tensile strength (TS) and elongation at break (EAB) reflect the rigidity and toughness of the composite film, respectively.

As shown in Figure 2A, when the dual-modified tapioca starch content was in the range of 1.0–3.0%, the TS of the composite film gradually increased from 5.1 MPa to 15.6 MPa, and the EAB gradually increased from 22.5% to 40.5% (1.0–2.0%) and then decreased to 32.3% (2.0–3.0%). The reason may be that with increasing dual-modified tapioca starch content, the arrangement of dual-modified tapioca starch molecules becomes more compact and orderly, and an increase in material density improves the mechanical properties of the composite film; however, the content of EAB decreases from 40.5% to 32.3% when the dual-modified tapioca starch content is 2.0% to 3.0%, possibly because the film thickness gradually increases and reaches the critical thickness for brittle transformation. Significant reductions in elongation were observed once the thickness of the material surpassed a critical threshold; this is similar to the findings of Supachok et al. [41], where the addition of thermoplastic starch to the composite film exceeded a critical value, restricting the mobility of the starch molecular chains and significantly decreasing the elongation at break of the nanocomposite materials (*p* < 0.05).

As shown in Figure 2B, when the glycerol content was in the range of 10% db–30% db, both the TS and EAB of the composite film first tended to increase but then decreased, with the TS increasing from 9.2 MPa to 13.7 MPa 10% db–20% db. It also decreased from 13.7 MPa to 11.3 MPa (20% db–30% db), and the EAB gradually increased from 4.2% to 10.6% (10% db–20% db) and then decreased to 8.4% (10% db–20% db). The reason may be that glycerol mainly plays the role of a plasticizer in film formation. In the range of 10% db–20% db, the C–O force of esterified tapioca starch increased, the formation of microcrystalline structures increased, and thus, the mechanical properties of the starch base film improved. However, excessive glycerol can lead to reconfiguration of the mesh structure of the composite film, resulting in structural loosening, which leads to deterioration of the mechanical properties of the film.

SiO_2_ was added to the film as an enhancement agent. As shown in Figure 2C, when the amount of SiO_2_ was within the range of 0.75% db–1.75% db, both the TS and EAB of the composite film first increased but then decreased, with the TS increasing from 18.14 MPa to 22.3 MPa (0.75% db–1.25% db). It then decreased from 22.3 MPa to 19.52 MPa (1.25% db–1.75% db), and the EAB gradually increased from 7.18% to 13.52% (0.75% db–1.25% db) and then decreased to 11.32% (1.25% db–1.75% db). The reason may be that after the addition of nanoparticles, intermolecular interactions between starch and glycerin–SiO_2_ occur, which improves the mechanical properties of the inner film within a certain range. However, when the addition amount is greater than 1.25%, the SiO_2_ cannot be fully dispersed evenly, and excessive SiO_2_ weakens the interaction between the plasticizer and starch molecules; this results in some aggregation behavior, which degrades the mechanical properties of the film.

After further optimization experiments, we selected 2% dual-modified tapioca starch, 2% chitosan solution, 20% db glycerol, and 1.25% db SiO_2_ to prepare the film matrix.

### 3.3. Antioxidant Capacity

**Figure 3:**
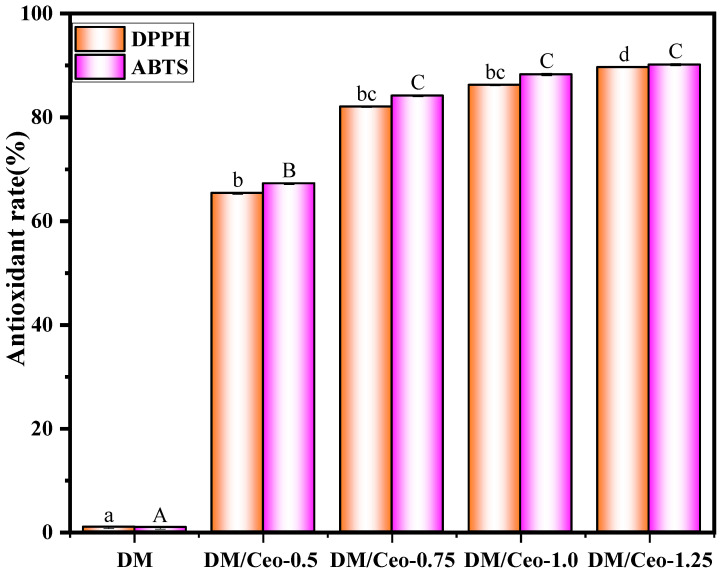
DPPH and ABTS free radical-scavenging rates of different composite film solutions.

To further improve the applicability of the film, Ceo was loaded into the base film with good mechanical properties to improve the antioxidant capacity of the composite film. As shown in Figure 3, in the DPPH radical-scavenging assay, the DM film solution basically has no antioxidant capacity, and the free radical clearance rate is only 0.9%, whereas in the DM/Ceo series of film solutions, the free radical clearance rates reach 65.38%, 82.06%, 86.25%, and 89.67%, respectively. In the ABTS radical-scavenging experiments, the Ceo-series film solutions demonstrated superior performance, with free radical-scavenging rates reaching 67.25%, 84.16%, 88.27%, and 90.15%, respectively. The results of the antioxidant experiments indicated that the free radical-scavenging capacity of the Ceo-series film solutions was positively correlated with the concentration of the Ceo emulsion, indicating that Ceo has great application potential in preventing food spoilage.

### 3.4. Wettability of Coating

The radical-scavenging rates for DPPH and ABTS reached 89.67% and 90.15%, respectively, with a 1.25% loading amount of the Ceo nanoemulsion. Considering the mechanical properties and light transmittance of the composite film, the DM/Ceo-1.25 film solution was finally selected for the preservation experiment of the green grapes. The TS of the DM/Ceo-1.25 film is 13.61 MPa, and the EAB is 52.02%. The transmittance at a wavelength of 600 nm is 60.01%. The transmittance values for the DM, DM/Ceo-0.5, DM/Ceo-0.75, and DM/Ceo-1.0 films are 58.6%, 59.2%, 59.6%, and 59.1%, respectively, all of which are lower than the value of the DM/Ceo-1.25 film.

**Figure 4:**
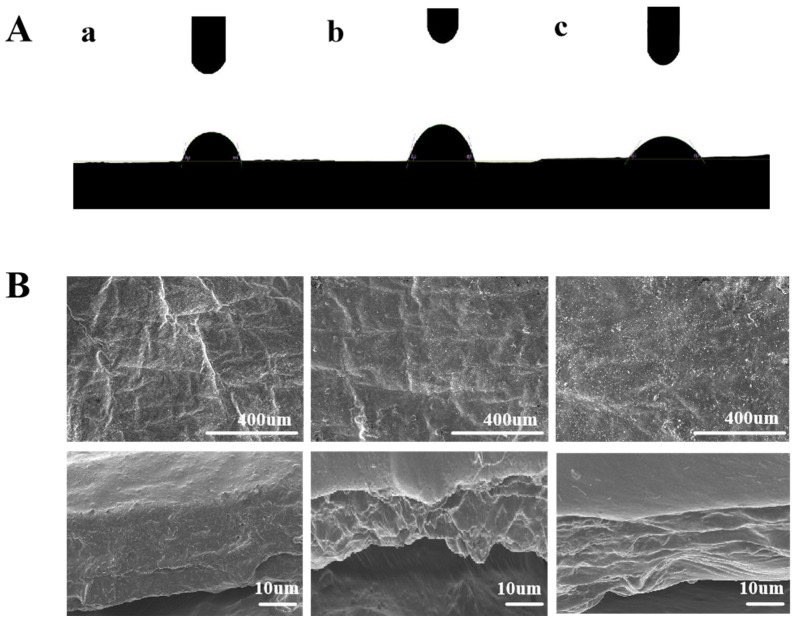
(**A**) Water contact angle of peels under different treatment methods (a: CK group. b: DM solution group. c: DM/Ceo-1.25 solution group). (**B**) Microscopic images of peels under different treatment methods (a–c: surface images of CK group, DM solution group, and DM/Ceo-1.25 solution group; d–f: cross-sectional images of CK group, DM solution group, and DM/Ceo-1.25 solution group).

Because the surface of the peel is hydrophobic, the adhesion ability of the coating on the hydrophobic surface of the peel is crucial to the coating effect. The formation of a lower contact angle between deionized water and the coating surface indicates that the coating possesses strong hydrophilicity, which facilitates its adhesion to the surface of the fruit [37]. The results are shown in Figure 4A. The water contact angles of the CK group, DM coating group, and DM/Ceo coating group were 75.0°, 65.5°, and 43.7°, respectively. Compared with the CK group and DM solution group, the DM/Ceo-1.25 solution group has relatively excellent coating characteristics, which enables it to spread faster. The contact angle formed between the DM/Ceo-1.25 coating and water is the smallest among the three groups examined, indicating that the hydrophilicity of the coating is greater than that of the other two groups. This result suggests that there is good adhesion between the coating and the fruit peel, facilitating the effective spreading of the coating solution on the surface of the fruit peel; the reason may be that Tween 80 is adsorbed at the interface, reducing the interfacial tension of water. This finding is consistent with the results of Liu et al. [42], where the lavender essential oil emulsion coating containing Tween 80 exhibited the highest wettability on banana peels compared with the other groups.

### 3.5. Morphology of the Green Grapes Coating

Figure 4B shows the scanning electron microscopy images of the surfaces (a–c) and cross-sections (d–f) of the CK group and the coated groups.

From the surface, the uneven mesh texture of the peel surface of the CK group can be clearly observed compared with the DM group and DM/Ceo group. The skin surface of the DM/Ceo coating group was flatter, indicating that the solution dispersion of the DM/Ceo film was better and easier to extend evenly. In addition, in the cross-sectional electron microscopy image, compared with the CK group, the DM group and DM/Ceo group successfully formed coatings on the surface of the pericarp, and the thicknesses of the two coatings were not much different.

### 3.6. Green Grapes Storage

#### 3.6.1. The Changes in Appearance of the Green Grapes During Storage and Microbiological Analyses

**Figure 5:**
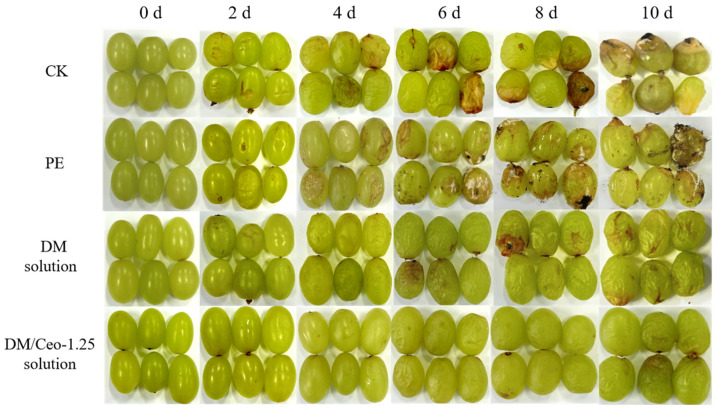
Changes in green grapes during 10 d storage under different treatment methods.

Appearance is an important factor for consumers when they choose produce. To determine the effect of the coating treatment on the appearance of the fruit, we observed the changes in the appearance of green grapes under four treatments (CK, PE, DM solution, and DM/Ceo-1.25 solution) over 10 days and recorded them with photos. As shown in Figure 5, the color of the grapes treated with the DM solution and DM/Ceo-1.25 solution from 0 to 4 d was brighter than that of the CK and PE groups, which is consistent with the color difference results in Table 3. On the 4th day, some of the coatings in the CK and PE groups began to corrupt, and from the 6th to 10th days, the number of rotten green grapes increased, and the degree of fruit deterioration in the PE group was more serious according to the naked eye observations because the water vapor transmission rate of the commercial PE film was low, and the water vapor generated by fruit respiration accumulated into water droplets, accelerating the propagation of microorganisms; this led to increased deterioration and even observable colony growth.

In the DM solution group, the fruit surface began to shrink from the 6th day, indicating that the water content began to decrease rapidly. With increasing time, by the 10th day, all of the experimental fruits had local disease spots. In the DM/Ceo-1.25 solution group, the storage period of the grapes was extended to the 10th day, and the shrinkage was also greater than that in the DM solution group, indicating that the coating more effectively inhibited the water loss of the grapes after the addition of Ceo and had an obvious antibacterial effect, extending the storage period of the grapes.

**Figure 6:**
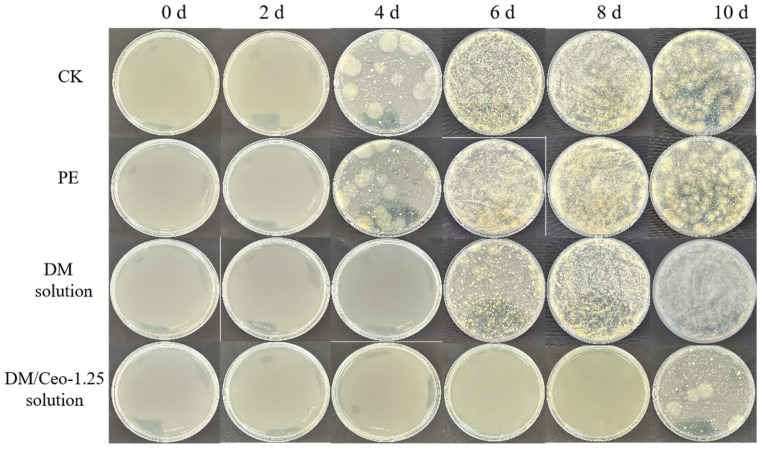
The total bacterial count of green grapes during 10 d storage under different treatments.

The microbial growth on the fruit during the 10 d storage period is shown in Figure 6, which is consistent with the results in Figure 5. During the 4th to 10th days, the number of molds and yeasts in the fruit increased, which was also the main reason for its severe spoilage. In the DM solution group, at 6 d, the microorganisms began to grow, causing black spots to appear on the surface of the green grapes. In the DM solution group, the microorganisms began to multiply on the 10th day, indicating that Ceo had an inhibitory effect on the growth of putrid bacteria, which was also the direct reason for the prolonged storage period of the green grapes in this group. Essential oils possess strong antibacterial and antioxidant properties, making them highly effective in extending the shelf life of fruits. Specifically, eugenol, which is rich in phenolic compounds, can interact with proteins upon incorporation into bacterial phospholipid membranes, thereby inhibiting their typical biological activities [43]. Previous research has demonstrated that polyvinyl alcohol films loaded with eugenol essential oil emulsion significantly reduce the microbial population on strawberry fruits during storage [44].

In addition, compared with those in the CK and PE groups, the storage conditions of the coated fruits in the two groups were greatly improved, which may have been due to the presence of the coating, which effectively blocked some of the exogenous microbial contamination and indicated that the coating also had a certain water retention effect because the shrinkage phenomenon of the green grapes was also alleviated in these two groups, which was consistent with the experimental results of the weight loss rate.

#### 3.6.2. Color, Hardness, Weight Loss Rate, Rot Rate

The color can directly reflect the freshness of green grapes, which largely determines whether consumers choose to buy them. As shown in Table 2, on day 0, the brightness (L*) of the peel in the DM solution group and the DM/Ceo-1.25 solution group was greater than that in the CK and PE groups because the coating was uniformly bright after drying. In addition, during the whole storage period (0–10 d), the brightness (L*) and greenness (a*) of the CK and PE groups decreased more than those of the coating group.

**Figure 7:**
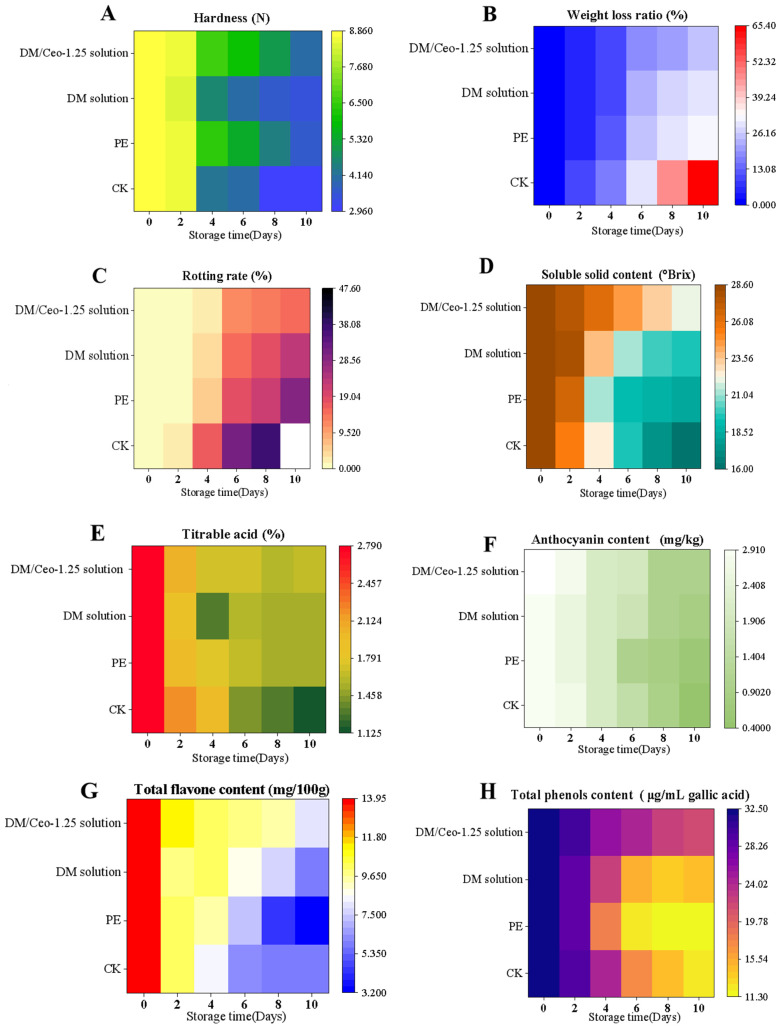
The physicochemical indexes of green grapes during storage period. (**A**) Hardness. (**B**) Weight loss rate. (**C**) Decay rate. (**D**) Soluble solids content. (**E**) Titratable acid. (**F**) Anthocyanin content. (**G**) Total flavone content. (**H**) Total phenol content.

The color retention of the DM/Ceo-1.25 solution group was relatively good, possibly because the Ceo in the coating inhibited the enzymatic browning reaction to a certain extent, and the oxidation rate of the active substances in the peel decreased. This finding was consistent with the experimental results of the contents of flavonoids (Figure 7G) and phenolics (Figure 7H) from the grapes in the different treatment groups. The loss of active substances was greatest in the CK group, whereas the loss in the DM/Ceo-1.25 solution group was relatively lower.

In addition, the yellowness (b*) of the two coating groups was greater at 0 d, possibly because the coating solution contained a chitosan solution. In the research by Alshehri et al. [45], similar findings were observed where the composite films incorporating chitosan showed a marked increase in the yellowness level (b*).

Hardness is a key parameter in evaluating fruit freshness. As shown in Figure 7A, the hardness of the green grapes in the CK and PE groups decreased faster than that in the coating group. The hardness of the green grapes in the DM/Ceo-1.25 solution group decreased the least, at only 4.82 N, whereas that in the CK group decreased the most, reaching 5.89 N. In general, water loss from a fruit is the direct cause of a decrease in hardness. The reduction in hardness makes fruits softer and more prone to deformation and damage, thus reducing their storage life and increasing waste. The results show that the coating exhibits an excellent water retention effect and maintains the hardness of the green grapes.

Weight loss is primarily related to transpiration during cellular metabolism. As shown in Figure 7B, owing to rapid water escape, the weight loss rate of the CK group reached 65.22% on day 10, while the weight loss rate of the PE group improved to 31.6% compared with that of the CK group. However, owing to the low water vapor transmission rate of PE, water accumulation resulted in the rapid propagation of microorganisms, which resulted in the fruit becoming black and soft; its rate of deterioration was the fastest among the four treatment groups (Figure 5). The weight loss rate of the DM/Ceo-1.25 solution group was only 25.3%, indicating that the coating has low water vapor permeability. It can prevent excessive volatilization of water from the green grapes, and the coating components form a network on the surface of the fruit that can accommodate moisture loss.

The commercial value of fruits is determined by their rot rate. As shown in Figure 7C, with increasing storage time, fruit decay occurred in the CK, PE, and DM groups on day 6, with decay rates reaching 30.2%, 18.3%, and 15.3%, respectively. The decay rate of the DM/Ceo-1.25 solution group was 15.2% on day 10, indicating that the DM/Ceo-1.25 solution group had a certain antibacterial effect, preventing microorganisms from infecting the fruits.

#### 3.6.3. Soluble Solids Content (SSC), Titratable Acidity (TA), Anthocyanin Content, TotalPhenol Content, Total Flavonoid Content

SSC and TA are important components of fruit flavor and quality. As shown in Figure 7D, with the extension of the storage period, the SSC showed an overall downward trend because physiological processes such as respiration and metabolism continuously consume organic acids in fruits. From 1 d to 10 d, the SSC of the CK group and PE group decreased by 12.6 °Brix and 9.8 °Brix, respectively, and that of the DM solution group and DM/Ceo-1.25 solution group decreased by 8.8 °Brix and 6.3 °Brix, respectively. These results indicated that the coating inhibited the physiological activity of the fruit extract to a certain extent to improve the fruit quality, and the DM/Ceo-1.25 solution had the best effect.

TA reflects the nutritional and physiological conditions of fruits and vegetables (Figure 7E) and is correlated with the SSC results, which also decreased over time. The DM/Ceo-1.25 solution effectively reduced the loss of TA during storage; the loss rate was only 1.125%, and the physiological quality was still better than that of the other groups on the 10th day. In addition, during the ripening process, the titratable acidity (TA) in all treatment groups rapidly decreased initially (0–6 days). After that, all fruits began to decline slowly, as reported by Gong et al. [46].

Anthocyanins, total phenols, and total flavonoids are important active nutrients in green grapes. As shown in Figure 7F, in all treatment groups, the anthocyanin content gradually decreased with the increase in storage time. This is consistent with the findings of Guo et al. [47]. The anthocyanin content in the CK and PE groups began to decline rapidly with the decay of the fruits from the 4th day, which rapidly decreased from 2.01 mg/kg to 0.404 mg/kg (10 d) in the CK group and from 1.99 mg/kg to 0.605 mg/kg (10 d) in the PE group. The concentration in the coating group decreased by 1.125 mg/kg and 1.059 mg/kg, respectively, and the rate of decrease was reduced.

In addition, as shown in Figure 7G, the phenolic content of the CK groups decreased rapidly during the 4th and 10th days of storage, respectively. The loss of phenolic content was due to the breakdown of the cell structure and senescence in the fruits. PE had no significant effect on the maintenance of phenolic content in the fruits, and the values were 17.715 μg/mL and 11.32 μg/mL after 4 d and 10 d, respectively. The DM/Ceo-1.25 solution effectively prevented the loss of phenolic substances and significantly extended the storage period of the green grapes. In the study by Teymoorian et al. [34], the content of phenolic compounds in fruits and vegetables coated with chitosan containing EO was higher than that in the control group. This suggests that essential oils may act as “signal compounds”, inducing an increase in antioxidants in the fruit tissue.

The content of flavonoids can represent the physiological activity of fruits. Figure 7H shows that the coating has a better effect on the retention of active substances. On the last day of storage, the flavonoid content in the DM solution group and the DM/Ceo-1.25 solution group remained at 5.91 mg/100 g and 8.025 mg/100 g, respectively, whereas in the CK group, this value decreased to 5.76 mg/100 g. The fruit had lost almost all of its nutritional value.

#### 3.6.4. Sensory Radar Map

**Figure 8:**
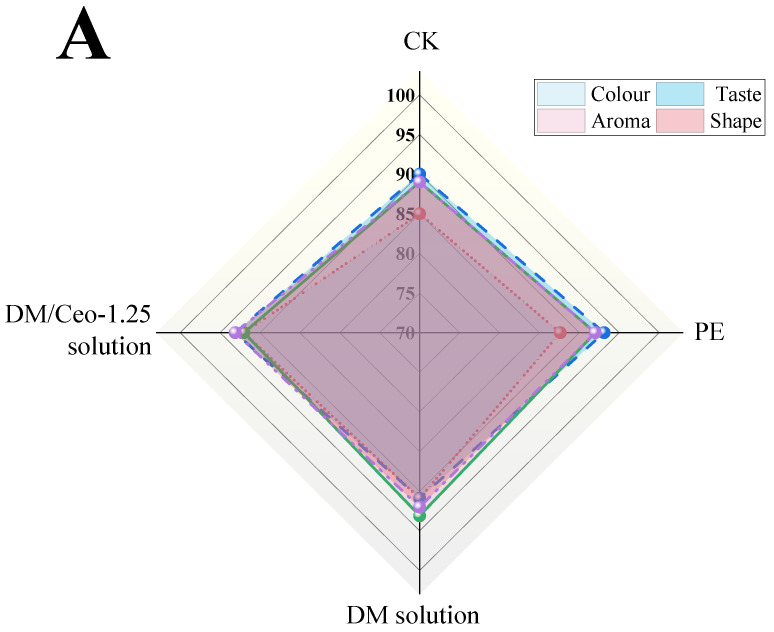

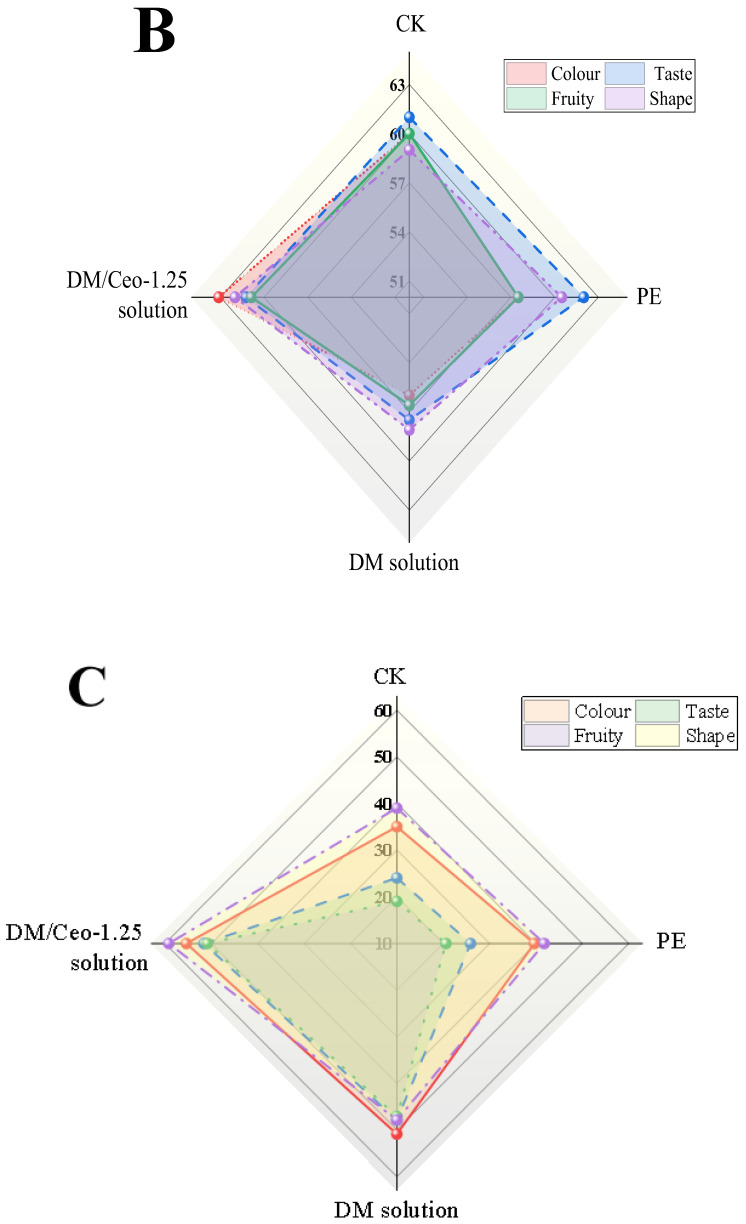
(**A**) Sensory scores of green grapes under different treatments at 0 d. (**B**) Sensory scores of green grapes under different treatments at 4 d. (**C**) Sensory scores of green grapes under different treatments at 10 d.

Figure 8A–C show the sensory radar maps of the green grapes subjected to the four different treatments on day 0, day 4, and day 10, and the green grapes were evaluated in terms of four aspects: color, taste, fruit aroma, and appearance. On day 0, there was no significant difference in scores among the four treatment groups in terms of color, taste, fruit aroma, and appearance. On day 4, the CK group and PE group presented the phenomenon of peel wrinkling, which greatly affected the scores of the sensory evaluation. In terms of appearance and shape, the scores were only 59 and 57, whereas the scores of the two coated groups were 77 and 82, respectively, for the fruits with a good appearance, complete shape, and no deterioration. On day 10, the four treatment groups all showed more or less rot, but the DM/Ceo-1.25 solution group was maintained relatively well. Owing to the good antibacterial action of Ceo, the fruit surface of this group did not show large black spots, and there was no obvious yellowing. This finding is consistent with the results of the color difference (Table 3). In the color index, the score of this group is 70, which still has certain commercial value.

#### 3.6.5. Correlation Analysis

**Figure 9:**
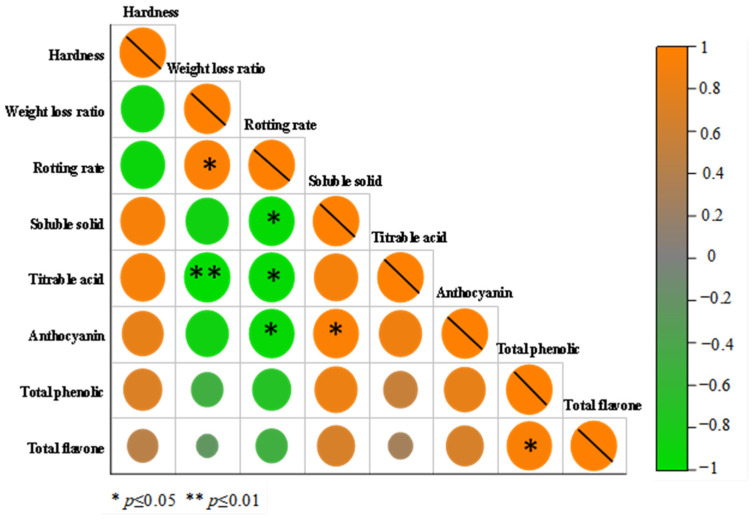
Correlation analysis.

Figure 9 displays the results of the Pearson correlation analysis between the indicators. This approach helps to understand the nature and strength of the linear relationship between two continuous variables. Correlation analysis has the potential to indicate whether there is an association between variables that might be worth exploring in more depth [27].

There was a significant positive correlation between the weight loss rate and decay rate (*p* < 0.05) and a significant negative correlation between the weight loss rate and titratable acid content (*p* < 0.01). These results indicated that water loss accelerated the decay of the fruit and worsened the basic quality characteristics of the fruit. With water loss, the hydrolysis of cell wall polysaccharides results in the production of a large amount of soluble solids, which increases the consumption of TA. There was a significant negative correlation between the decay rate and the soluble solid content, titratable acid, and anthocyanin contents (*p* < 0.05), indicating that fruit decay directly led to a rapid decline in the nutritional value of the green grapes.

## 4. Conclusions

In this study, a dual-modified tapioca starch-based coating loaded with Ceo emulsion, which has good physical and antibacterial properties, was successfully prepared. Compared to the blank group and PE group, the DM/Ceo-1.25 solution could extend the storage period of the green grapes.

Furthermore, the decline in nutritional indicators was mitigated, and the quality indicators were well maintained. Additionally, in the sensory evaluation on the tenth day, the DM/Ceo-1.25 coating group scored 70 points, significantly outperforming the control group (CK) with 59 points, and the PE group with 57 points. It is likely that this improvement is due to the action of the antimicrobial substance, clove essential oil, and the ability of the coating to regulate the respiration rate of the fruit. Subsequent investigations should explore this matter in greater depth. Our study provides a safe alternative for fruit preservation.

## Figures and Tables

**Table 1:** The formulations of different solutions. DM solution: DM/CS solution. DM/Ceo-1.25 solution: dual-modified tapioca starch/CS/1.25 wt% clove essential oil nanoemulsion solution.

Elements	Samples		
	CK	DM Solution	DM/Ceo-1.25 Solution
Double-modified tapioca starch (g)	-	2	2
Chitosan solution (mL)	-	100	100
Glycerin (g)	-	0.4	0.4
CEO (g)	-	-	2.5
Nano-SiO_2_ (g)	-	0.05	0.05
Water (mL)	100	100	100

**Table 2:** Particle size, zeta, and PDI of different emulsions.

Samples (Ceo/Tween 80/Water)	Particle Size (nm)	Zeta (mV)	PDI
1:1:8	190.14 ± 0.91 ^a^	−32.3 ± 0.66 ^a^	0.188 ± 0.021 ^a^
1:2:7	396.95 ± 2.18 ^b^	−23.5 ± 0.45 ^b^	0.511 ± 0.015 ^b^
2:1:7	255.01 ± 1.36 ^ab^	−21.9 ± 0.73 ^bc^	0.267 ± 0.024 ^a^
3:1:6	458.67 ± 2.04 ^c^	−23.3 ± 0.25 ^bc^	0.138 ± 0.030 ^a^
4:1:5	531.17 ± 2.49 ^c^	−25.6 ± 0.68 ^c^	0.274 ± 0.017 ^a^

The results are expressed as the mean ± standard error. Different lower-case letters denote significant differences within different samples (*p* < 0.05).

**Table 3:** Peel color index of different treatment groups during 10 d storage period.

Storage Time (Days)	Color	CK	PE	DM Solution	DM/Ceo-1.25 Solution
0	L*	33.32 ± 2.21 ^b^	34.16 ± 1.96 ^c^	36.92 ± 2.23 ^c^	37.47 ± 0.94 ^c^
	a*	−2.53 ± 0.23 ^a^	−2.72 ± 0.31 ^a^	−3.19 ± 0.61 ^a^	−3.12 ± 0.24 ^a^
	b*	8.75 ± 0.04 ^a^	8.73 ± 0.68 ^a^	9.31 ± 1.34 ^a^	9.77 ± 0.57 ^a^
2	L*	30.75 ± 0.98 ^b^	31.71 ± 1.12 ^b^	33.65 ± 1.96 ^b^	35.71 ± 1.22 ^b^
	a*	−1.87 ± 0.34 ^a^	−1.82 ± 0.12 ^a^	−2.55 ± 0.34 ^a^	−2.27 ± 0.26 ^a^
	b*	11.98 ± 0.21 ^a^	11.58 ± 1.61 ^b^	10.93 ± 1.46 ^a^	11.77 ± 1.34 ^a^
4	L*	29.47 ± 0.56 ^b^	30.15 ± 0.98 ^b^	33.58 ± 1.64 ^b^	34.56 ± 1.69 ^a^
	a*	−1.53 ± 0.21 ^a^	−1.63 ± 0.34 ^a^	−2.78 ± 0.27 ^a^	−2.37 ± 0.13 ^a^
	b*	12.22 ± 0.69 ^b^	12.03 ± 1.14 ^b^	13.13 ± 0.67 ^b^	12.91 ± 1.65 ^a^
6	L*	27.54 ± 0.96 ^a^	28.69 ± 0.65 ^a^	31.33 ± 1.02 ^a^	34.51 ± 1.37 ^a^
	a*	1.03 ± 0.43 ^b^	−0.56 ± 0.13 ^b^	−1.10 ± 0.41 ^b^	−2.13 ± 0.17 ^a^
	b*	13.33 ± 0.71 ^b^	13.07 ± 1.28 ^b^	14.81 ± 1.05 ^b^	13.88 ± 1.28 ^b^
8	L*	26.98 ± 0.36 ^a^	27.99 ± 0.87 ^a^	30.99 ± 0.62 ^a^	33.9 ± 0.91 ^a^
	a*	2.34 ± 0.64 ^c^	1.63 ± 0.24 ^b^	−0.69 ± 0.35 ^b^	−1.76 ± 0.34 ^a^
	b*	13.38 ± 1.31 ^b^	14.12 ± 0.69 ^b^	15.16 ± 0.67 ^b^	13.89 ± 0.31 ^b^
10	L*	26.05 ± 0.68 ^a^	27.18 ± 0.76 ^a^	29.53 ± 0.45 ^a^	32.55 ± 1.04 ^a^
	a*	2.70 ± 0.81 ^c^	1.67 ± 0.27 ^b^	−0.26 ± 0.30 ^b^	−1.30 ± 0.27 ^a^
	b*	13.91 ± 1.26 ^b^	15.68 ± 0.06 ^b^	15.31 ± 1.36 ^b^	14.39 ± 0.45 ^b^

CK group: uncoated, washed with deionized water, and dried. PE group: wrapped with commercially available plastic wrap. DM group: coated with DM film solution. DM/Ceo-1.25 group: coated with DM/Ceo-1.25 film solution. The results are expressed as the mean ± standard error. Different lower-case letters denote significant differences within different samples (*p* < 0.05). L* represents the lightness of a color, with higher values indicating brighter shades. The a* value indicates the position of a color on the red-green axis, where positive values suggest a tendency towards red and negative values indicate a lean towards green. The b* value represents the location of a color on the yellow-blue axis, with positive values indicating a yellowish cast and negative values suggesting a bluish tint.

## Data Availability

The original contributions presented in the study are included in the article; further inquiries can be directed to the corresponding authors.

## Abbreviations

Clove essential oil (Ceo); chitosan (CS); dual-modified tapioca starch (DM); DM/CS coating solution (DM coating solution); dual-modified tapioca starch/CS/clove essential oil (DM/Ceo); polyethylene wrap (PE) group; polymer dispersity index (PDI); potato dextrose agar (PDA); titratable acid (TA); tensile strength (TS); elongation at break (EAB).
